# Characterization of soil organic matter in perhumid natural cypress forest: comparison of humification in different particle-size fractions

**DOI:** 10.1186/1999-3110-54-56

**Published:** 2013-11-15

**Authors:** Jenn-Shing Chen, Tay-Lung Chung, Guanglong Tian, Chih-Yu Chiu

**Affiliations:** 1 Yung-Ta Institute of Technology & Commerce, Linlo, Pingtung 90942 Taiwan; 2 Biodiversity Research Center, Academic Sinica, Nankang, Taipei 11529 Taiwan; 3 Environmental Monitoring and Research Division, Monitoring and Research Department, Metropolitan Water Reclamation District of Greater Chicago (MWRD), Lue-Hing R&D Laboratory, 6001 W. Pershing Road, Cicero, IL 60804 USA

**Keywords:** CP/MAS ^13^C NMR, Perhumid forest, Particle-size fraction, Soil organic matter, Humification degree

## Abstract

**Background:**

The *Chamaecyparis* forest is a valuable natural resource in eastern Asia. The characteristics of soil humic substances and the influence of environmental factors in natural *Chamaecyparis* forests in subtropical mountain regions are poorly understood. The study site of a perhumid *Chamaecyparis* forest is in the Yuanyang Lake Preserved Area in northcentral Taiwan. We collected samples from organic horizons (Oi, Oe and Oa) and from the surface horizon (O/A horizon) at the summit, footslope and lakeshore to characterize the composition of the soil organic matter. Samples of organic horizons were dried and ground, and those of the O/A horizon were passed through wet sieving for different particle-size fractions before analysis. The C chemical structure in the samples was determined with CP/MAS ^13^C NMR spectra.

**Results:**

The ratios of alkyl-C/*O*-alkyl-C and aromaticity increased with decomposition of litter from the Oi, Oe, to Oa horizon. The ratio of alkyl-C/*O*-alkyl-C also increased from coarse (> 250 μm) to very fine (< 2 μm) particle fractions, which indicates increased humification of soil organic matter (SOM) in the fine-sized fractions. However, aromaticity tended to decrease with decreasing particle size, so it may not be useful in evaluating SOM humification of different particle-size fractions.

**Conclusions:**

The humification degree of the samples from O horizons and different particle-size fractions of the O/A horizon showed no gradient change with change in topography. This prevalent slow decomposition of organic matter in these perhumid climate conditions may narrow the difference in humification from the summit to lakeshore.

**Electronic supplementary material:**

The online version of this article (doi:10.1186/1999-3110-54-56) contains supplementary material, which is available to authorized users.

## Background

Humus substances are the most recalcitrant and major fraction of soil organic matter (SOM). The humus in soils has beneficial effects on plant nutrient supply, soil structure and water-holding capacity. Because of high stability, humus benefits carbon storage in soils. However, decomposition of humus releases CO_2_, which may contribute to increased atmospheric CO_2_ level and the greenhouse effect (Piccolo, 1996).

*Chamaecyparis* cypress forest is a valuable natural resource in eastern Asia for its high-quality timber. As well, the accumulation of non-decomposed organic matter on the forest floor provides a critical buffer to retain water and prevent soil erosion with heavy rainfall in montane areas. The dead timber and thick SOM might store a previously underestimated large C pool. Several decades ago, before large-scale logging, *Chamaecyparis* forest was widely distributed in cloudy montane areas in Taiwan, at about 800 to 2800 m a.s.l.

Much effort has been invested in investigating soil properties and fertility management of Japanese hinoki cypress (*Chamaecyparis obtusa*) plantations (e.g., Inagaki et al. 2008;2011). By contrast, characteristics of humic substances under natural *Chamaecyparis* forests in this subtropical montane area are not well known because most of the preserved natural *Chamaecyparis* forests are located in steep and remote areas with poor access to roads. The Chi-Lan Mountain contains one of the few natural preserved *Chamaecyparis* forests in northcentral Taiwan.

Podzolic soils are commonly found under *Chamaecyparis* forests in the cold and humid subalpine region in Taiwan (Chiu et al. 1999; Jien et al. 2010a, [Bibr CR17][Bibr CR19]). The extremely high acidity and soil moisture in such undisturbed *Chamaecyparis* forest results in low diversity of soil bacterial communities (Lin et al. 2010,2011) as well as low decomposition of organic matter and humification degree of humic acids (Chung et al. 2012). Our previous studies revealed that the topography and intrinsic properties of different fractions of organic matter in a mountain lake environment affect the distribution and migration of organic substances (Chen and Chiu 2000). The degree of humification of SOM decreased slightly from the summit to lakeshore, and the relatively low degree of humification was due to high precipitation and acidity (Chung et al. 2012).

Particle-size fractionation has been widely used to study the physical and chemical properties and decomposition of SOM (Joliveta et al. 2006; Muñoz et al. 2009). Our previous study indicated that the humification degree of SOM increased from coarse to fine fraction size in humid subalpine soils (Chen and Chiu 2003). We hypothesized that SOM humification might be hampered differently by varied decomposition with different particle-size fractions, with possible interactions of SOM characteristics, particle-size fractions and soil moisture conditions. We aimed to clarify the differences in organic matter composition in various particle-size fractions as affected by topography in a *Chamaecyparis* forest.

## Methods

### Study site and sampling

The Yuanyang Lake ecosystem (24°35’N, 121°24’E) is located in the northcentral part of Taiwan from 1670 to 2169 m a.s.l. This region is a cloud forest system because of annual precipitation of 4000 mm and annual mean temperature 13°C, with a temperate perhumid climate. This area was selected as a representative long-term ecological study site of Taiwan. The vegetation is dominated by Hinoki cypress (*C. obtusa*) and Taiwan false cypress (*C. formosensis*) with an understory evergreen broadleaf shrub (*Rhododendron formosanum*).

The forest soils were characterized as Albaquult, Dystrochrept and Histosol in the summit, footslope and lakeshore regions, respectively (Chiu et al. 1999; Chen and Chiu 2000). The organic matter at the summit and footslope is mor type, which is a thick mat of non-decomposed to partially decomposed O horizons, and humic peat type at the lakeshore with continuous water saturation, for a moderately decomposed class of peat characterized by 1/3 to 2/3 recognizable plant fibers. Soil at the summit is well drained, whereas that at the footslope and lakeshore is poorly drained. In particular, the lakeshore, located about 1.5 m above the lake, frequently experiences inundation of water during the monsoon season.

We selected 3 representative pedons along a topographic sequence at the summit, footslope and lakeshore. At each study site, a pit was excavated for describing macromorphological soil characteristics and for collecting soil samples according to standard procedures (Soil Survey Staff 2006). The toposequence for clay mineralogical characterization in this study site has been described elsewhere (Pai et al. 2007). Organic horizons (i.e., Oi, Oe and Oa) were classified and collected on the basis of rubbed fiber content that can be identified by eyes and fingers.

In addition, we collected the O/A horizon for comparing particle-size fractions. Three replicate subsamples were collected from each topographic position with use of a soil auger 8 cm in diameter and 10 cm deep before being bulked together. Non-decomposed and partially decomposed plant litter was removed before sampling.

### Physical and chemical analyses

Soil samples first underwent low-energy sonication and then were separated into different particle-size fractions by a combination of wet sieving and continuous flow centrifugation. Four particle-size fractions, including coarse (> 250 μm), medium (53–250 μm), fine (2–53 μm), and very fine (< 2 μm), were separated (Chen and Chiu 2003). All fractionated samples were freeze-dried and stored. Samples collected from Oi, Oe, and Oa horizons were dried at 70°C and ground for analyses.

Solid-state CP/MAS ^13^C NMR determination involved use of a Bruker DSX 400 MHz instrument operating at 100.46 MHz and spin rate 7 kHz. The 0.5-g soil sample was used each time. Acquisition parameters were contact time, 1 ms; pulse delay time, 1 s; and spectra plotted region, 0 to 200 ppm (Chen and Chiu 2003; González-Pérez et al. 2008; Novak and Smeck 1991). About 10,000 scans were collected for samples (Quideau et al. 2001). The C functional groups were determined by the following chemical-shift areas (Novak and Smeck 1991; Chen and Chiu 2003; Jien et al. 2011): alkyl-C signals, 0 to 50 ppm; *O*-alkyl-C signals, 50 to 90 ppm; di-*O*-alkyl C signals, 90 to 110 ppm; aromatic-C signals, 110 to 165; and carboxyl-C signals, 165 to 190 ppm. The methoxyl-C of the chemical shift signal appeared at 56 ppm and overlapped with the *O*-alkyl-C signals (Knicker 2000). The signal intensities in the respective chemical shift regions are expressed as a percentage of the total spectra area. The relative contents of different chemical structures were calculated from the area under the spectra. To compare each spectrum for samples, we chose the peak of carboxyl C as the standard peak and standardized the signals by adjusting the peak area to the same size for each sample. Then, we compared the peaks of different functional groups in terms of relative percentage of each functional C group. The area for each functional C group peak was calculated by use of Topspin software for the NMR instrument.

Chemical shift regions were used to calculate the ratio of alkyl-C/*O*-alkyl-C (Baldock et al. 1997) and aromaticity (Hatcher et al. 1981; Almendros et al. 2000) as follows:

1 $$\mathrm{Ratio}\;\mathrm{of}\;\mathrm{alkyl}-C/O-\mathrm{alkyl}-C=\frac{\mathrm{alkyl}-C\;\mathrm{peak}\;\mathrm{area}\;\left(0-50\mathrm{ppm}\right)}{\left(O-\mathrm{alkyl}-C+\mathrm{di}-O-\mathrm{alkyl}\;C\right)\;\mathrm{peak}\;\mathrm{area}\left(0-50\mathrm{ppm}\right)}$$

2 $$\mathrm{Aromaticity}=\frac{\mathrm{aromatic}-C\;\mathrm{peak}\;\mathrm{area}\;\left(110-165\mathrm{ppm}\right)}{\left(\left(\mathrm{alkyl}-C+O-\mathrm{alkyl}-C+\mathrm{di}-O-\mathrm{alkyl}\;C+\mathrm{aromatic}\;C\right)\mathrm{peak}\;\mathrm{area}\left(0-165\mathrm{ppm}\right)\right)}$$

The above ratios were used to evaluate the humification degree and decomposition of SOM.

### Chemical analysis

The total C (TC) and total N (TN) contents in the samples from O horizons and samples of particle-size fractions from the O/A horizon were determined after combustion in a Nitrogen Analyzer (NA1500 Series 2, Fisons, Italy).

## Results

### Chemical characteristics and ^13^C NMR analyses

The pH of the soil samples was strongly acidic (3.5 ~ 3.8) and not very different in profiles. The TC was higher at the footslope and summit than the lakeshore (Table 1). The trend for TN content was similar to that for TC content. The C/N ratio decreased with depth in the 3 horizons (Oi, Oe, and Oa): highest at Oi and lowest at Oa. In addition, the C/N ratio increased from the summit to lakeshore, particularly for the Oi horizon.

**Table 1:** **The chemical properties of partially decomposed materials collected at various organic horizons in 3 topographic positions of the Yuanyang Lake ecosystem in Taiwan**

Pedons	Soil type	Organic horizon	Depth (cm)	pH (H_2_O)	TC^a^(%)	TN^b^(%)	C/N ratio
Summit	Albaquult	Oi	8-10	3.7	35.2	1.4	25.1
		Oe	3-8	3.5	49.2	2.1	23.4
		Oa	0-3	3.5	49.2	2.2	22.3
Footslope	Dystrochrept	Oi	11-15	3.8	54.2	1.8	30.1
		Oe	3-11	3.5	51.2	2.1	24.3
		Oa	0-3	3.3	47.7	2.3	20.7
Lakeshore	Histosol	Oi	24-33	3.5	36.3	1.1	33.0
		Oe	11-24	3.4	18.7	0.6	31.1
		Oa	0-11	3.5	17.7	0.7	23.9

^a^Total carbon.

^b^Total nitrogen.

The TC and TN content in the samples for the O/A horizon increased with decreasing particle size (Table 2). The topographic position did not affect TC and TN content in different fractions of the O/A horizon samples. The C/N ratios gradually decreased with decreasing particle size at the 3 topographic positions.

**Table 2:** **Total C, N content and C/N ratio in each particle-size fraction of soils collected from the O/A horizon at 3 topographic positions**

Topographic position	Particle-size (μm)	TC^a^(%)	TN^b^(%)	C/N
Summit	Coarse (>250)	44.4	1.1	39.0
	Medium (250 ~ 53)	43.8	1.5	29.5
	Fine (53 ~ 2)	44.7	2.0	21.9
	Very Fine (< 2)	46.9	2.4	19.6
Footslope	Coarse (>250)	41.9	1.9	22.0
	Medium (250 ~ 53)	45.8	2.0	22.4
	Fine (53 ~ 2)	38.4	1.8	21.8
	Very Fine (< 2)	47.3	2.3	20.7
Lakeshore	Coarse (>250)	42.0	1.5	27.7
	Medium (250 ~ 53)	47.0	1.7	28.1
	Fine (53 ~ 2)	49.1	1.9	26.5
	Very Fine (< 2)	47.5	1.8	26.5

^a^Total carbon.

^b^Total nitrogen.

**Figure 1:**
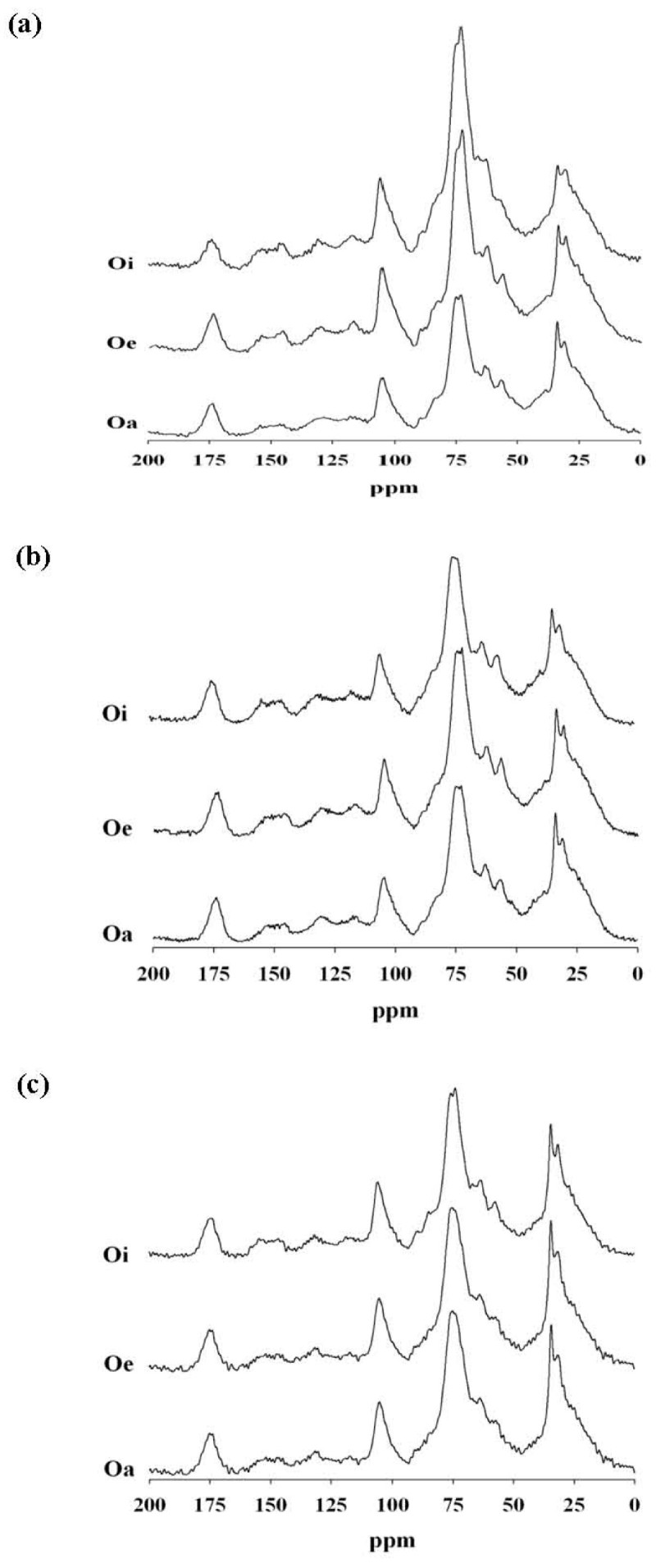
**CP/MAS** ^**13**^ **C NMR spectra for partially decomposed plant materials in 3 organic horizons (Oi, Oe, and Oa) at the (a) summit, (b) footslope, and (c) lakeshore.**

Figure 1 shows the characteristics of CP/MAS ^13^C NMR spectra of partially decomposed plant materials at the 3 horizons. The relative distribution of integrated peak areas of chemical shift regions in the CP/MAS ^13^C NMR for different carbon functional groups of organic matter from each horizon was in the order of alkyl-C > *O*-alkyl-C > aromatic-C > di-*O*-alkyl-C > carboxyl-C at all 3 topographical positions (Table 3). The content of alkyl-C and carboxyl-C increased with depth at the footslope and lakeshore but not at the summit. By contrast, the content of *O*-alkyl-C and di-*O*-alkyl-C slightly decreased with depth, but that of aromatic-C and carboxyl-C slightly increased with depth at all positions.

**Table 3:** **Relative distribution of integrated peak areas of chemical shift regions in the CP/MAS** ^**13**^ **C NMR spectra of partially decomposed materials collected from 3 organic horizons at 3 topographic positions**

Topographic position	Horizon	C functional group (%)					A/ ***O***-A ratio^a^	Aromaticity^b^
		alkyl	***O-*** alkyl	di- ***O***-alkyl	aromatic	carboxyl		
Summit	Oi	34.1	30.5	15.0	13.7	6.7	0.75	0.15
	Oe	33.7	30.5	13.5	16.4	5.9	0.77	0.17
	Oa	33.5	30.0	12.5	16.2	7.8	0.79	0.18
Footslope	Oi	26.7	31.6	15.4	18.3	8.0	0.57	0.20
	Oe	28.2	30.9	14.9	18.2	7.8	0.61	0.20
	Oa	27.8	30.1	14.4	19.9	7.7	0.62	0.22
Lakeshore	Oi	30.9	26.4	15.4	19.9	7.4	0.74	0.21
	Oe	31.0	26.1	14.6	20.4	7.8	0.76	0.22
	Oa	31.3	25.9	14.2	20.7	7.9	0.78	0.22

^a^alkyl-C peak area (0–50 ppm)/( (*O*-alkyl-C + di-*O*-alkyl C) peak area (50–110 ppm) ).

^b^aromatic C peak area (110–165 ppm)/ ((alkyl-C + *O*-alkyl-C + di-*O*-alkyl C + aromatic C) peak area (0–165 ppm)).

### ^13^C NMR analyses of particle-size fractions of O/A horizon

**Figure 2:**
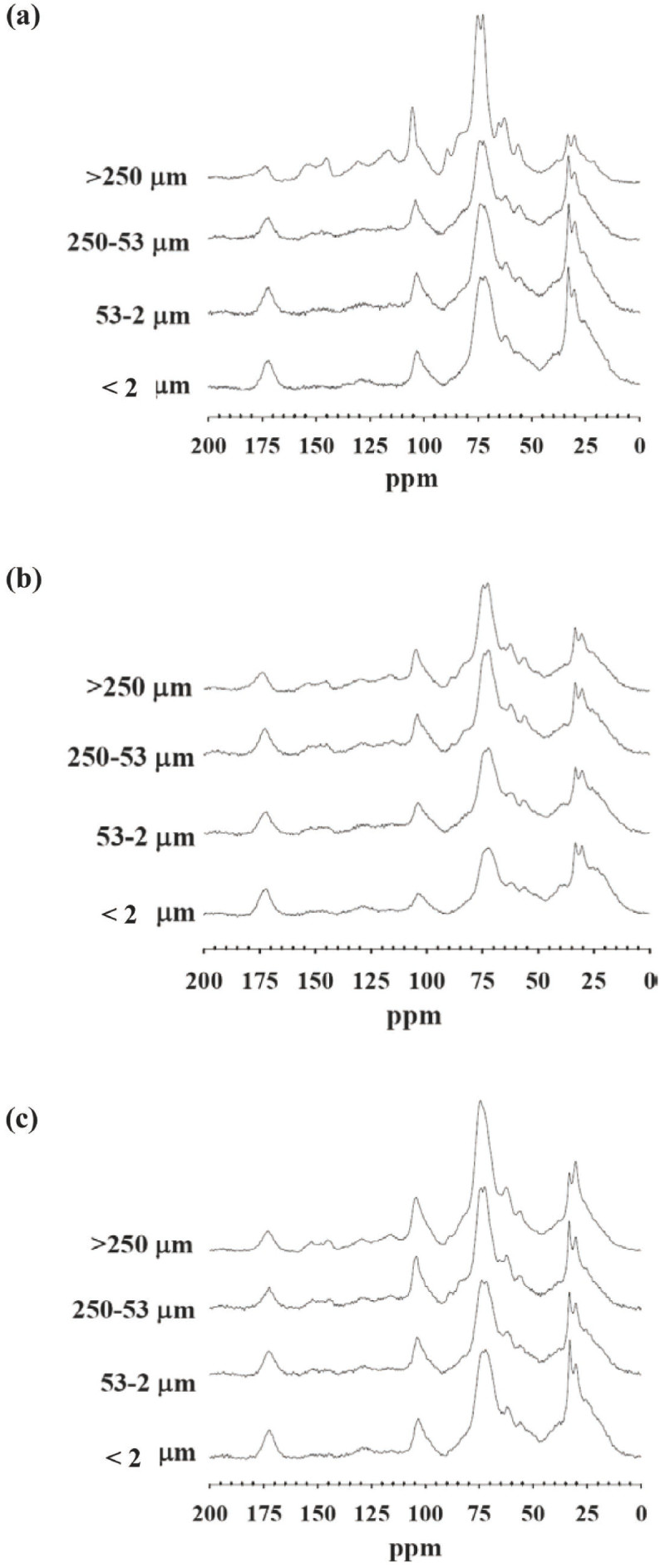
**CP/MAS** ^**13**^ **C NMR spectra for soil organic matter in different particle-size fractions from the O/A horizon at the (a) summit, (b) footslope, and (c) lakeshore.**

Coarse (> 250 μm) particle size was the dominant (61.0 ~ 83.3%) fraction, followed by medium (250–53 μm) and fine fractions (53–2 μm), with the minimum (1.1 ~ 2.1%) being the very fine fraction (< 2 μm) (Table 4). The ^13^C NMR spectra for organomineral (O/A horizon) particle-size fractions (Figure 2) and the results of peak area integrations (Table 5) from the summit to lakeshore demonstrated that fraction size largely affected the C structure. The distribution of C functional groups showed a predominance of *O*-alkyl-C and alkyl-C groups and a relatively low content of aromatic-C groups in the samples. The amount of *O*-alkyl-C, di-*O*-alkyl-C and aromatic-C decreased with decreasing particle size, and alkyl-C content increased with decreasing particle size. The aromatic-C content was lower with fine than coarse particle size. The alkyl-C/*O*-alkyl-C ratio and aromaticity responded to particle size differently, with an increase for alkyl-C/*O*-alkyl-C ratio and a decrease in aromaticity from coarse to fine particle size. In comparing different topographic positions, *O*-alkyl-C and di-*O*-alkyl-C contents were highest at the lakeshore, particularly in coarse and medium fractions (Table 5). By comparison, alkyl-C content was lowest at the lakeshore, particularly in the finest particle-size fraction.

**Table 4:** **Particle-size distribution of soils collected from the O/A horizon (0–10 cm) at 3 topographic positions**

Topographic	Particle size (μm)			
position	Very fine (< 2)	Fine (2–53)	Medium (53–250)	Coarse (>250)
Summit	1.6	19.8	17.5	61.0
Footslope	2.1	15.1	18.2	64.5
Lakeshore	1.1	6.0	9.5	83.3

Data are percentages.

**Table 5:** **Relative intensities of the CP/MAS** ^**13**^ **C NMR spectra in different particle-size fractions isolated from the O/A horizon (0–10 cm) in 3 topographic positions**

Topographic	Particle-size	C functional groups (%)					A/ ***O***-A ratio^a^	Aromaticity^b^
position	(μm)	alkyl	***O-*** alkyl	di- ***O***-alkyl	aromatic	carboxyl		
Summit	Coarse (>250)	28.4	42.9	9.9	13.7	5.1	0.54	0.14
	Medium (250 ~ 53)	31.2	42.4	9.3	11.8	5.3	0.60	0.12
	Fine (53 ~ 2)	36.9	41.8	8.2	8.3	4.7	0.74	0.09
	Very fine (<2)	41.2	41.7	7.1	5.3	4.6	0.84	0.06
Footslope	Coarse (>250)	29.5	41.5	9.5	13.5	6.0	0.58	0.14
	Medium (250 ~ 53)	31.8	42.2	8.9	11.5	5.5	0.62	0.12
	Fine (53 ~ 2)	34.9	39.4	8.5	11.4	5.8	0.73	0.12
	Very fine (<2)	41.5	35.7	6.8	9.3	6.7	0.98	0.10
Lakeshore	Coarse (>250)	28.8	45.3	10.2	11.1	4.6	0.52	0.12
	Medium (250 ~ 53)	28.4	44.4	10.6	11.8	4.9	0.52	0.12
	Fine (53 ~ 2)	34.7	41.8	8.7	9.2	5.6	0.69	0.10
	Very fine (<2)	38.3	42.1	7.8	6.9	4.9	0.77	0.07

^a^ alkyl-C peak area (0–50 ppm)/( (*O*-alkyl-C + di-*O*-alkyl C) peak area (50–110 ppm) ).

^b^ aromatic C peak area (110–165 ppm)/ ((alkyl-C + *O*-alkyl-C + di-*O*-alkyl C + aromatic C) peak area (0–165 ppm)).

## Discussion

### Chemical composition

Samples from 3 organic horizons of the Yuanyang Lake ecosystem predominantly consisted of organic matter. The high content of C in these horizons was consistent with that in other perhumid forests (Mafra et al. 2007; Schawe et al. 2007). Poor drainage and flooding in riparian soils diminishes the decomposition of SOM but improves the denitrification by microorganisms (Mafra et al. 2007). Thus, we found lower TN content and higher C/N ratio at the lakeshore than footslope and summits (Table 1). The C/N ratio varied markedly by particle-size fraction at the summit but not greatly at the footslope and lakeshore (Table 2). High total organic C content throughout the fractions indicates the accumulation of organic matter in the soil. In addition, high percentages (61% to 83%) of samples were in the coarse fraction with decomposing plant residues (Table 4), which suggests the retardation of organic matter decomposition in such perhumid climate conditions.

#### Carbon functional groups in different particle-size fractions

We found *O*-alkyl-C and di-*O*-alkyl-C as the dominant components in the entire soil fraction, which can probably be used by microorganisms during humification processes (Keeler et al. 2006). The region of aromatic-C includes phenolic-C, derived from lignin and tannin, and bacterial resynthesized compounds consisting of alkyl-C and carboxyl-C (Mahieu et al. 1999; Mathers et al. 2000; Ussiri and Johnson 2003; López et al. 2008).

Physical fractionation of soil according to particle size followed by chemical, biological, and physical analyses of fractions is a powerful tool in process-oriented SOM research (Mao et al. 2007). Particle-size fractioning allows for separating SOM pools of varying degrees of microbial alteration and mineral association (Joliveta et al. 2006). The SOM in the coarse fraction primarily consisted of labile plant residues, whereas microbial biomass is supposed to be concentrated in the very fine fraction (Zech et al. 1996; Kimetu et al. 2008). Thus, SOM associated with very fine fractions tends to be more aliphatic than does whole SOM (Mao et al. 2007; Jiménez et al. 2008; Muñoz et al. 2009). We found low C/N ratio (Table 2) and high alkyl-C content (Table 5) in very fine particle-size fractions, which suggests the loss of easily decomposable carbohydrates and selective preservation of inherently recalcitrant materials during the plant residue decomposition (Mathers et al. 2000; Wagai et al. 2008; Rovira et al. 2009).

The *O*-alkyl-C content in coarse and medium fractions was higher at the lakeshore than at the summit and footslope. In particular, peaks at 56, 63, 84 and 89 ppm (*O*-alkyl-C) were completely diminished from coarse (>250 μm) to very fine (<2 μm) at the summit, with only a trace amount at the footslope and lakeshore (Figure 2). The *O*-alkyl-C content might be greater at the lakeshore than other positions and was universally lower in fine than coarse particles (Table 5), as was found in our previous study of subalpine forest and grassland soils (Chen and Chiu 2003). Thus, the spectra of the coarse fraction (>250 μm) resembled that of decomposing materials (Figures 1 and 2), which resulted from most of the soils being in a coarse fraction (Table 4).

#### Humification degree of organic horizons and particle-size fractions of the O/A horizon

The humification degree and characteristics of SOM could be evaluated by ratio of alkyl-C/*O*-alkyl-C and aromaticity. The high ratio of alkyl-C/*O*-alkyl-C and aromaticity indicates the high humification degree of SOM in the soils (Baldock et al. 1997; Almendros et al. 2000; Chen et al. 2004; Mueller and Koegel-Knabner 2009). The alkyl-C/O-alkyl-C ratios of the Oi, Oe, and Oa horizons in the 3 positions increased with increasing soil depth (Table 3), which indicates a higher humification degree in the Oa horizon. We found no typical pattern for alkyl-C/O-alkyl-C ratios in O horizons along topographical positions (Table 3), which indicates restrained decomposition and humification because of low temperature and high annual precipitation. The results for aromaticity were similar to those for alkyl-C/O-alkyl-C (Table 3), which could reflect the humification state of SOM. The influence of litter input on changes in remnant masses of each carbon during the early humification processes showed that mass loss rate of aromatic C in humified litter is higher in cypress than cedar (Ono et al. 2009,2011). Our previous studies showed that humification degree of HAs, determined by E4/E6 ratio (Chen et al. 2001a, b) and by ^13^C NMR (Chung et al. 2012) differed little by topographic position of the study site.

The ratio of alkyl-C/*O*-alkyl-C increased with decreasing particle-size fraction (Table 5), so high stability in the fine fraction is due to the protection by clay minerals (Wagai et al. 2008) and to the chemical resistance of the alkyl-C structure to decomposition. The humification degree in the medium and fine fractions of the O/A horizon samples was similar to that in the O horizons in the 3 studied positions, which reflects the chemical properties in SOM being strongly dominated by the distribution of particle-size fractions. The ratios of alkyl-C/*O*-alkyl-C in each particle-size fraction with topographic change under the *Chamaecyparis* forest were similar in value and variation and were lower than in *Tsuga* forest soils (Chen and Chiu 2003). Thus, the relatively low proportion of alkyl-C/*O*-alkyl-C ratios is due to the poor decomposition under perhumid climate conditions.

By comparison, aromaticity findings were opposite to those for ratios of alkyl-C/*O*-alkyl-C in particle-size fraction. Aromaticity decreased with decreasing particle size (Table 5). The present results agree with the suggestions of Hempfling et al. (1987) and Almendros et al. (2000 (2000) questioning the increase in aromaticity during humification in soils. The results of Baldock *et al.* (1997) also showed that aromatic C contents tended to decrease with decreasing particle size, which suggests that increased extent of decomposition was not accompanied by an increase in aromatic C content. If aromatic C content did not accumulate with decomposition, an increase in aromaticity should not be used as an indicator of the extent of decomposition.

## Conclusions

The distribution of particle-size fractions in soil is a critical factor in determining the chemical composition of SOM. ^13^C NMR analysis of soil particle-size fractions revealed that the C structure of the SOM changes with different particle-size fraction. The accumulation of recalcitrant C compounds in the fine particle-size fraction was contributed by alkyl-C rather than aromatic-C. The ratio of alkyl-C/*O*-alkyl-C indicates increasing SOM humification with decreasing particle size. The humification degree showed no gradient change pattern in different topographical positions. The effect of topography on the decomposition and humification of organic matter in the *Chamaecyparis* forest was apparently overshadowed by the slow decomposition of organic matter.

## Authors’ original submitted files for images

Below are the links to the authors’ original submitted files for images. Authors’ original file for figure 1 Authors’ original file for figure 2
