# Supplementary figures and images for: Characterization of soil organic matter in perhumid natural cypress forest: comparison of humification in different particle-size fractions
Source: Bot Stud. 2013 Nov 15;54:56. doi: 10.1186/1999-3110-54-56 (PMC5430335; doi:10.1186/1999-3110-54-56)

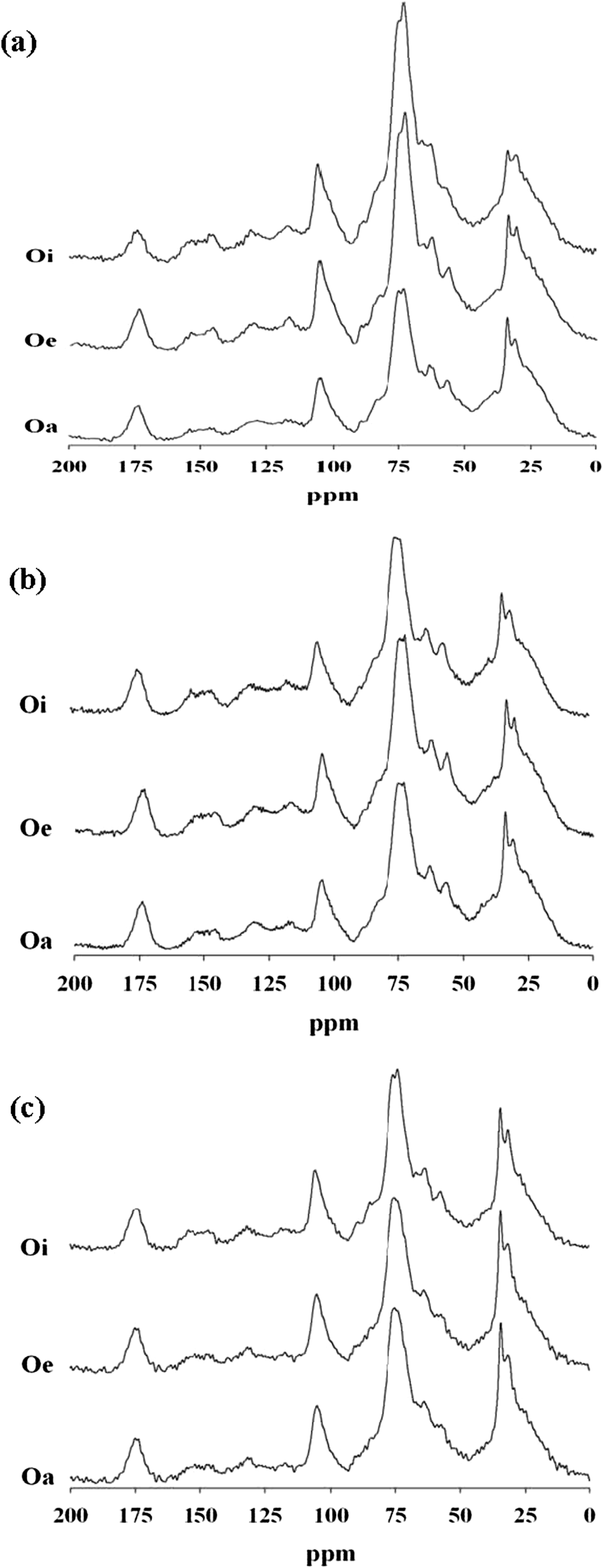

Supplement: Supplementary file 1 — Authors’ original file for figure 1 [file 40529_2012_100_MOESM1_ESM.tiff]

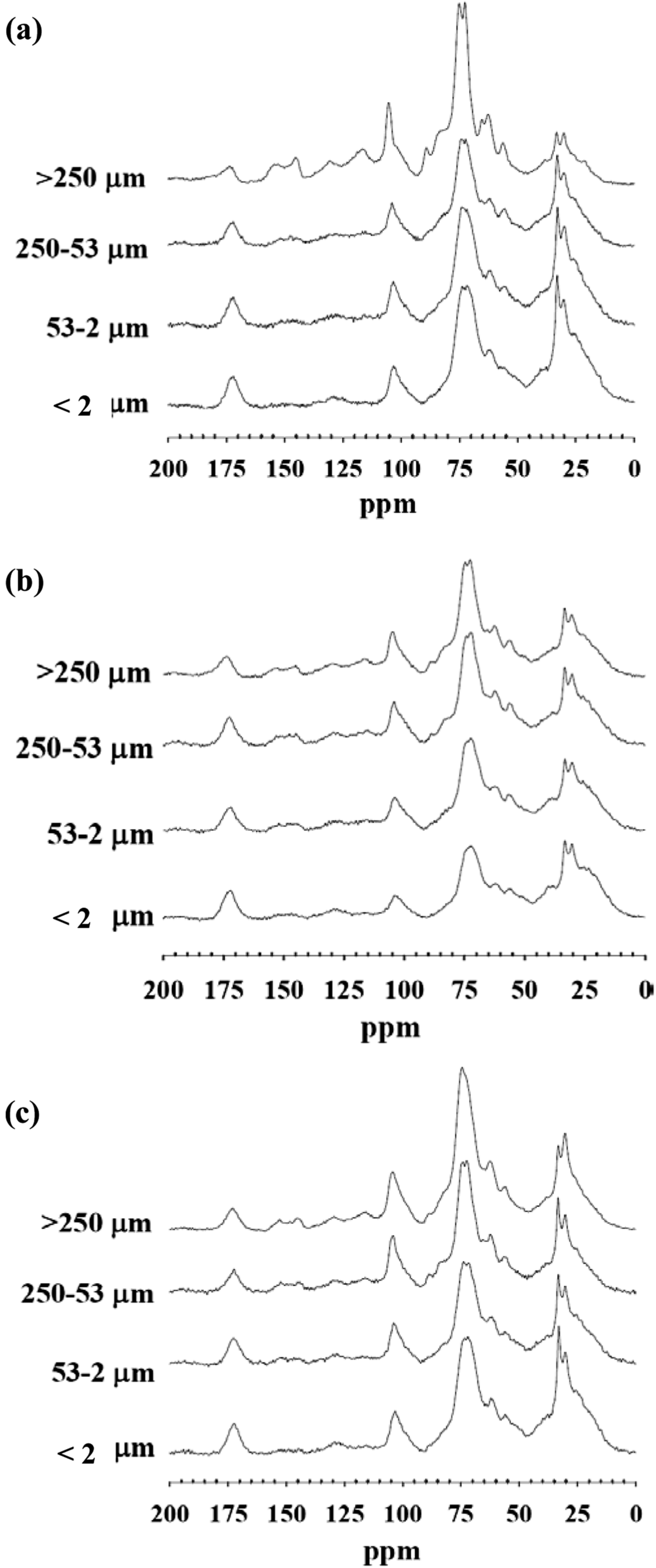

Supplement: Supplementary file 2 — Authors’ original file for figure 2 [file 40529_2012_100_MOESM2_ESM.tiff]
